# Tregs in Autoimmunity: Insights Into Intrinsic Brake Mechanism Driving Pathogenesis and Immune Homeostasis

**DOI:** 10.3389/fimmu.2022.932485

**Published:** 2022-06-30

**Authors:** Kyle J. Bednar, Jee Ho Lee, Tatiana Ort

**Affiliations:** Bioscience Immunology, Research and Early Development, Respiratory & Immunology, BioPharmaceuticals R&D, AstraZeneca, Gaithersburg, MD, United States

**Keywords:** T-regulatory cell, immune-mediated diseases, cytokine, cell therapy, pathways

## Abstract

CD4^+^CD25^high^Foxp3^+^ regulatory T-cells (Tregs) are functionally characterized for their ability to suppress the activation of multiple immune cell types and are indispensable for maintaining immune homeostasis and tolerance. Disruption of this intrinsic brake system assessed by loss of suppressive capacity, cell numbers, and Foxp3 expression, leads to uncontrolled immune responses and tissue damage. The conversion of Tregs to a pathogenic pro-inflammatory phenotype is widely observed in immune mediated diseases. However, the molecular mechanisms that underpin the control of Treg stability and suppressive capacity are incompletely understood. This review summarizes the concepts of T_reg_ cell stability and T_reg_ cell plasticity highlighting underlying mechanisms including translational and epigenetic regulators that may enable translation to new therapeutic strategies. Our enhanced understanding of molecular mechanism controlling Tregs will have important implications into immune homeostasis and therapeutic potential for the treatment of immune-mediated diseases.

## Introduction

Regulatory T (Treg) cells, a subset of helper T cells, are pivotal in supporting immune tolerance and preventing autoimmunity (1, 2). Forkhead box P3 (Foxp3) is a master transcription factor required for Treg function and development. Mutations in Foxp3 gene led to impaired Treg functions, which resulted in excessive autoimmunity in multi-organs in both human and mouse (3, 4). On the contrary, forced ectopic expression of Foxp3 induces the differentiation into Treg-like cells from conventional T cells (5). Foxp3^+^ Treg cells also express high levels of IL-2R (CD25) and CTLA-4 on their surface, which are well-characterized Treg-lineage markers and associated with suppressive activity (1). There are two sub-populations of Treg cells based on origins. Thymic Treg (tTreg) cells are naturally occurring in the thymus by self-antigens and IL-2 while induced Treg cells in the periphery (pTreg) are differentiated by TGF-β plus IL-2. Both Treg subsets have similar functions, but tTregs are relatively more stable than pTregs due to the differential demethylation of TSDR (Treg-specific demethylated region) in Fopx3 gene locus (6). Treg cells can execute their functions through multiple mechanisms including immune-suppressive cytokine expression, metabolite deprivation, cytotoxic molecule production and surface inhibitory receptor expression making them an attractive cell therapy target for the treatment of autoimmunity. Dysregulation of Treg development, homeostasis, function, or stability would result in defective immune tolerance and eventually trigger autoimmunity. Indeed, a number of studies have reported that defects in maintenance of Treg number or function are associated with diverse human autoimmune diseases including type 1 diabetes (7–10), systemic lupus erythematosus (11–14), rheumatoid arthritis (15, 16), inflammatory bowel diseases (17–19), Sjögren’s syndrome (20, 21), and multiple sclerosis (22). Besides, there is evidence that Treg cells can be reprogramed to Th17 (19, 23) or Th1 cells (24), implying this pathogenic Treg conversion can further promote excessive inflammation in patients. Thus, it is a key mechanism limiting Treg conversion as well as sustaining the immune-regulatory function for Treg cell adoptive transfer therapy.

## Cytokines and Treg Stability

It is a question of interest how Treg cells maintain their functional activity in physiological environments. Treg-specific fate-mapping reporter mice enabled to study Treg stability have demonstrated that Treg cells maintained their immune-suppressive activity under both normal and inflammatory conditions (25). However, many other studies have reported that Treg cells often lost Foxp3 along with reduced suppressive function under various inflammatory conditions (26–28). Such Treg instability has been identified in human patients or animal models with inflammatory diseases such as IBD, RA, and SLE (29).

Since Treg cells are present in various organs including lymphoid and peripheral tissues, they often encounter the cytokine milieu in the inflammatory microenvironment. These inflammatory cytokines can control Treg development, function and plasticity at the local tissues during immune responses. For example, IL-2 is indispensable for Treg development and proliferation by controlling STAT5 signaling pathway (30, 31). IL2R/STAT5 signals also confers Treg lineage stability with retained function (32, 33). TGF-β signaling in combination with activation of IL-2R signaling pathway promotes Treg differentiation from naïve CD4+ T cells (34). The downstream molecules such as Smad3 and STAT5 of TGF-β and IL-2 signaling pathways, respectively, play a critical role for Treg generation and Foxp3 stability *via* binding to conserved non-coding sequences (CNS) in the Foxp3 gene locus (35). TGF-β signaling also supports Treg stability by prevention of conversion into IFN-γ-expressing cells through inhibition of IL-12R expression (36). These results indicated that IL-2 and TGF-β are pivotal in Treg homeostasis in preventing autoimmunity. Both cytokines can play a role in TET protein expression or recruitment to the CNS2 locus. TET proteins will be discussed later as important modulator of demethylation and Tregs stability, but because of this role they also suppress Treg plasticity to acquire Teff responses (37, 38).

Tumor necrosis factor α (TNF-α) plays an important role in pathogenesis of autoimmune diseases. Several studies revealed that TNF-α modulates immune regulation by affecting Treg expansion and/or stability (39–41). Treg cells preferentially express TNFR2 on the surface compared to conventional CD4^+^ T cells (42), and TNFR2-mediated signaling prevents methylation on Foxp3 gene locus, leading to maintained Treg stability (40). Indeed, TNFR2-deficient Treg cells displayed Foxp3 loss with increased methylation in the proximal promoter of Foxp3 gene, leading to reduced suppressive function, and TNFR2-deficient mice further showed severe disease progression with reduced number of Treg cells in collagen-induced arthritis model (41). TNFR2 designed ankyrin repeat proteins (DARPins) have been used to target TNFR2 in Tregs as an antibody mimetic. TNFR2 DARPins showed increased i-κB degradation in human primary Tregs and increased NF-κB activation in a Jurkat reporter cell line, which could lead to decreased suppressive functions. However, conflicting literature exists showing TNFR2 agonistic antibody or TNF-α mutant protein selectively binding to TNFR2, expand highly potent regulatory Tregs (43, 44). Moreover, TNF-α together with IL-6 enables human Treg proliferation with sustained function and stability *ex vivo* (45). Anti-TNF therapy has been employed, but some patients treated with anti-TNF therapy develop disease exacerbation (46), implying that these therapies may hinder immune-suppressive function of Treg cells. However, TNFR2-mediated signaling dampened Treg activity in some studies (42, 47, 48), suggesting Treg function regulated by TNF-TNFR2 signaling should be controlled for drug development of autoimmune diseases.

IL-6 is known to be an inflammatory cytokine in several autoimmune diseases including systemic lupus erythematosus, arthritis, and experimental autoimmune encephalomyelitis. IL-6-mediated STAT3 signaling promotes Th17 cell differentiation, while it suppresses Foxp3^+^ Treg polarization (49). Beyond Treg differentiation, IL-6 also induces Foxp3 instability and impaired Treg function through regulation of STAT3 under the inflammatory disease condition (50). IL-6/STAT3 signaling hinders the suppression of Foxp3 on RORγt, which is a key transcription factor for IL-17 expression in Th17 cells. Interestingly, Blimp-1 can inhibit the methylation of Foxp3 gene locus by suppression of Dmnt3a expression in response to IL-6. Blimp-deficient mice displayed loss of Foxp3 in Tregs cells accompanied with increased severity of central nervous system diseases (51). It has been identified that Treg conversion into Th17-like cells in RA patients (52) supporting IL-6 drives instability of Treg cells.

IL-1 family cytokines have been investigated to have impacts on Treg homeostasis and stability. For example, IL-1β can inhibit Treg development by activation of HIF-1A/mTOR pathway (53). IL-1β also promotes the conversion of Treg cells into IL-17-producing cells albeit intact Foxp3 expression unlike IL-6 (54). However, IL-33, another IL-1 family cytokine, maintains Treg stability under inflammatory conditions by suppressing expression of IL-17 or IFN-γ (54, 55). Additionally, IL-33 together with IL-18 induces amphiregulin expression by Treg cells, which is critical for tissue repair upon viral infection (56).

Interferon gamma (IFN-γ) is a critical cytokine for both innate and adaptive immune responses. NK cells and Th1 cells predominantly produce IFN-γ, but the aberrant expression of IFN-γ can trigger various autoimmune diseases. T-bet activation induced by IFN-γ drives expression of CXCR3, a homing receptor, in Treg cells like Th1 cells. The ablation of T-bet+ Treg cells enhances Th1-dependent autoimmunity (57) indicating this distinctive subset of Treg cells specifically restrains conventional Th1 cells. In contrast, IFN-γ can promote Treg conversion into IFN-γ-expressing Th1-like cells, which further cause excessive inflammation or anti-tumor responses (58, 59). The accumulated HIF-1α by VHL-deficient Treg cells induces IFN-γ expression with Foxp3 loss in Treg cells. These observations indicate that pathogenic plasticity of Treg cells may increase under inflammatory hypoxic condition or changes in metabolism.

## FOXP3 Stability Through Functional and Phenotypic Screens

To better understand how FOXP3 and CTLA4 are regulated either at steady state or under inflammatory conditions, multiple medium-through put screens have been run in recent years. Notably, small molecule and secretome phenotypic screens have identified novel regulators that stabilize or destabilize Tregs at steady state. In both instances, the iQue flow system was used as to measure the protein expression of FOXP3 and CTLA4 in primary human Tregs as surrogates for stability and function. For the small molecule screen, a compound library consisting of diverse chemical matter including known epigenetic, kinase, and other target class modulators were screened (60). This resulted in the discovery of intracellular proteins euchromatic histone-lysine N-methyltransferase (EHMT2) and glycogen synthase kinase 3 alpha/beta (GSK3a/b) as positive regulators of FOXP3 and CTLA4 expression.

EHMT2 is a histone methyltransferase that catalyzes the methylation of histone 3 lysine 9 (H3K9). Epigenetic regulation of FOXP3 at Treg-specific demethylated regions (TSDR) plays an important role in Treg stability and function. Studies in murine Tregs using a small molecule inhibitor of a DNA methyltransferase (azacytidine) also induced stabilization of FOXP3 expression adding confidence to the small inhibitor in human Tregs (61). Murine studies using CRISPR-Cas9 deletion of ten-eleven translocation protein (TET), promoted partial demethylation of TSDR CNS2 region of FOXP3, resulting in stable FOXP3 expression *in vivo* (62). However, these results were not corroborated in a separate study in which they utilized the TET1 catalytic domain fused to an enzymatically dead Cas9. This resulted in targeting TET1 to the CNS2 locus causing cytosine demethylation at ~30% of CNS2, this level of demethylation did not result in stability of FOXP3. However, more efficient demethylation targeting may be required (63). TET protein(s) are well-studied proteins for murine Treg stability and has direct links to CNS2. Hydrogen sulfide (H2S) promotes TET1 and TET2 protein expression resulting in subsequent DNA demethylation by recruitment of the TET proteins by IL-2 and TGF-β signaling (64). Further, TET proteins have a major role in Treg development in the thymus and deletion of TET2/TET3 markedly compromises Foxp3 expression and stability. Further, vitamin C potentiates TET2/3 activity to promote demethylation in TGF-β induced Tregs, again highlighting it’s important role in Treg stability (65, 66). The interplay between methylation and acetylation is also becoming clearer in its importance for Treg stability. In a recent study, they used Gene Ontology analysis to identify Foxp3 regulators which are highly enriched in genes encoding subunits of the spt-Ada-Gcn5 acetyltransferase (SAGA) chromatin-modifying and switch/sucrose non-fermentable (SWI/SNF) chromatin remodeling complexes. They proceeded to use CRISPR/Cas9 to reveal an important role for *Brd9*, a key protein in the ncBAF complex, in Treg function. *Brd9* regulated Foxp3-dependent transcriptional programs reducing Tregs suppressive capacity. *In vivo Brd9* deletion reduced Treg suppressor activity in a T-cell transfer model of colitis while increasing anti-tumor immune responses in an MC38 colorectal cancer model (67). Histone deacetylases also are implicated in Treg biology through multiple mechanisms, including histone deacetylase 7 (HDAC7) and histone acetyltransferase (HAT) p300. HDAC inhibitors increased FOXP3 expression through the regulation of protein and gene expression. Not only was FOXP3 protein elevated, but this also led to functional effects in both humans and mice, showing greater ability to suppress Teff cell responses (68, 69). HAT p300 was deleted through CRISPR/Cas9 and lead to the discovery that p300 targeted the promotor locus of FOXP3 and stabilized protein levels even under inflammatory conditions (63). Targeted small molecule inhibitors or CRISPR deletion of proteins that drive epigenetic changes could be key in driving stable Tregs both in the periphery or for Treg cell therapy.

GSK3 is a growth signaling-sensitive kinase which is represented by nonredundant isomers, alpha and beta. GSK3 is involved in diverse biological functions in lymphocytes. In T-cells and Tregs it has been previously validated to increase expression of IL-10, stabilize FOXP3, regulate activation and exhaustion, while also being able to promote pTreg generation (70–75). Inhibition of GSK3β stabilized the expression of β-catenin which is reported to induce expression of FOXP3 and improve Treg survival (76). In agreement, with the role of GSK3β inhibition acting as a stabilizer of FOXP3, it was demonstrated that a GSK3β activator which results in GSK3β- phosphorylation resulted in subsequent ubiquitination and degradation of FOXP3 by E3 ligase β-transducing repeat containing protein (74, 77). Ubiquitination of histones at the Foxp3 locus and/or of the protein itself is important to regulate expression. Members of the SAGA complex is also involved in the deubiquitinating of FOXP3, including ubiquitin specific peptidase 22 (Usp22). Deletion of Usp22 drives reduction in Foxp3 transcript levels and through increased ubiquitin at the chromatin level ultimately leading to Foxp3 degradation. *In vivo* loss of Usp22 resulted in lower suppressive activity of Tregs, correlating degradation of Foxp3 to functional activity (67, 78). E3 ubiquitin ligases induced by inflammation and responsible for ubiquitination of Foxp3 include, Rnf20 (78), TRAF6 (79) and STUB1 (80). These all show key roles in causing degradation of FOXP3 in mouse and human Tregs. The ubiquitin pathway thus represents an interesting area of research for targeting through genetic manipulation.

The secretome high-throughput screen assessed 575 secreted proteins for the upregulation of FOXP3 and CTLA4. As described above secreted proteins can have diverse effects on Treg cell function and stability. This identified four proteins, growth differentiating factor-7 (GDF-7), acidic prostate phosphatase (PAP), interferon alpha-7 (IFNα-7), and IL-10 (a well-known cytokine regulating Treg function) (81). GDF-7 belongs to the TGF-β superfamily of proteins. Signaling *via* TGF-β is well documented to support Treg stability and function. GDF-7 function has not been previously well described for Tregs, however it binds to a heterodimeric receptor consisting of type 1 and type 2 heterodimers (82). Another GDF protein, GDF-15, has increased serum levels in patients with hepatocellular carcinoma and correlated to increased numbers of FOXP3^+^ Tregs. It was further demonstrated to play an important role in the tumor microenvironment to drive pTreg development and enhance the suppressive function of tTregs by downregulating STUB1, an E3 ligase that mediates FOXP3 protein degradation (83). Further examination of TGF-β superfamily members for the control of Tregs will help elucidate the roles of this superfamily on Treg biology, including GDF-7. PAP was originally described in patients with prostate cancer, hence its name, and is used as a diagnostic biomarker. Interestingly, PAP has shown nucleosidase activity *in vitro*, converting AMP into free adenosine. Adenosine is a well described molecule which possess immuno-suppressive properties. The molecules which convert ATP/ADP and AMP into free adenosine are upregulated on Tregs, CD39 and CD73, respectively. Free adenosine then acts *via* the ADORA2A/A2a receptor. This receptor has been described to be important in Treg numbers and function in murine models (84, 85). Finally, IFNα-7 is less well described then the molecules above to have positive effects on FOXP3 levels. Further, there are conflicting reports on how type I interferons may affect regulatory T-cells and is an area of research which needs further explored (86, 87).

Interestingly other well-known inflammatory cytokines in the secretome screen were not found to affect FOXP3 and CTLA4 expression. Explanations for this could be naïve Tregs were used at steady state, change in receptor expression after expansion, or while Foxp3/CTLA4 levels were unchanged, their suppressive capacity or phenotype was altered which was undetected in this medium-through put screen. Evidence for the importance of other signaling receptors and known transcription factors have come to light from CRISPR/Cas9 experiments. Recently, additional evidence confirming years of research into the importance of IL-2 for Tregs was demonstrated *via* CRISPR/Cas9. IL-2RA deletion resulted in the loss of STAT5 signaling and Treg suppressive function. Similarly, IL-6R alpha subunit showed reduction in STAT3 activation, suggesting the possibility of genetically engineered Tregs to be resistant to IL-6 mediated Treg instability (88). While transcriptional regulation is not a focus of this review, a paper by Schumann et al. entitled “functional CRISPR dissection of gene networks controlling human regulatory T cell identity” pressured Tregs under inflammatory cytokines while using CRISPR/Cas9 to reveal important transcription factors regulating Treg instability in the inflammatory settings. This screen highlighted four such factors including IRF4, FOXO1, STAB1, and HIVEP2 (89). It will be pivotal to expand on this seminal work by generating data outside of transcription factors or to a whole genome wide screen to further elucidate the gene networks responsible for stability and function of Tregs for the treatment of autoimmune diseases.

## Treg Expansion and Advances in Treg Cell Therapy

Human Treg biology has been a challenge due to their relatively low frequency in peripheral blood, lower proliferation rates and loss of function, survival and phenotypic stability in long-term cultures and after freeze thaw (90–92). Recently, many of these challenges have been overcome, opening the field of human Treg biology to be used in adoptive cell therapy, high-through put screens and novel genome editing techniques like CRISPR (clustered, regularly interspaced, short palindromic repeats)/Cas9 (CRISPR associated protein 9) (93–95). These advances on top of the decades of fundamental research support the use of Tregs in adoptive cell therapy. Polyclonal Treg cell-based therapies have previously been shown to be safe and well tolerated for the treatment of autoimmunity and transplant rejection, although the efficacy was limited (96), therefore new approaches are being developed to increase their efficacy in particular genetic engineering. Briefly, using genetically engineered Tregs in adoptive cell therapy could help to overcome the short-comings with current autologous polyclonal Treg methods by increasing Treg stability in inflammatory environments, enhancing their function, or targeting Tregs to tissues or sites of inflammation, these aspects are reviewed elsewhere (97–99). Genetically modified Tregs have recently advanced to clinical trials in transplantation: chimeric antigen receptor (CAR)-Tregs genetically engineered to specifically target HLA-A2 and drive Treg activation to prevent immune-mediated rejection in HLA-A2 mismatched kidney and liver transplants (NCT04817774 and NCT04838171) (100–102).

## Discussion

**Figure 1:**
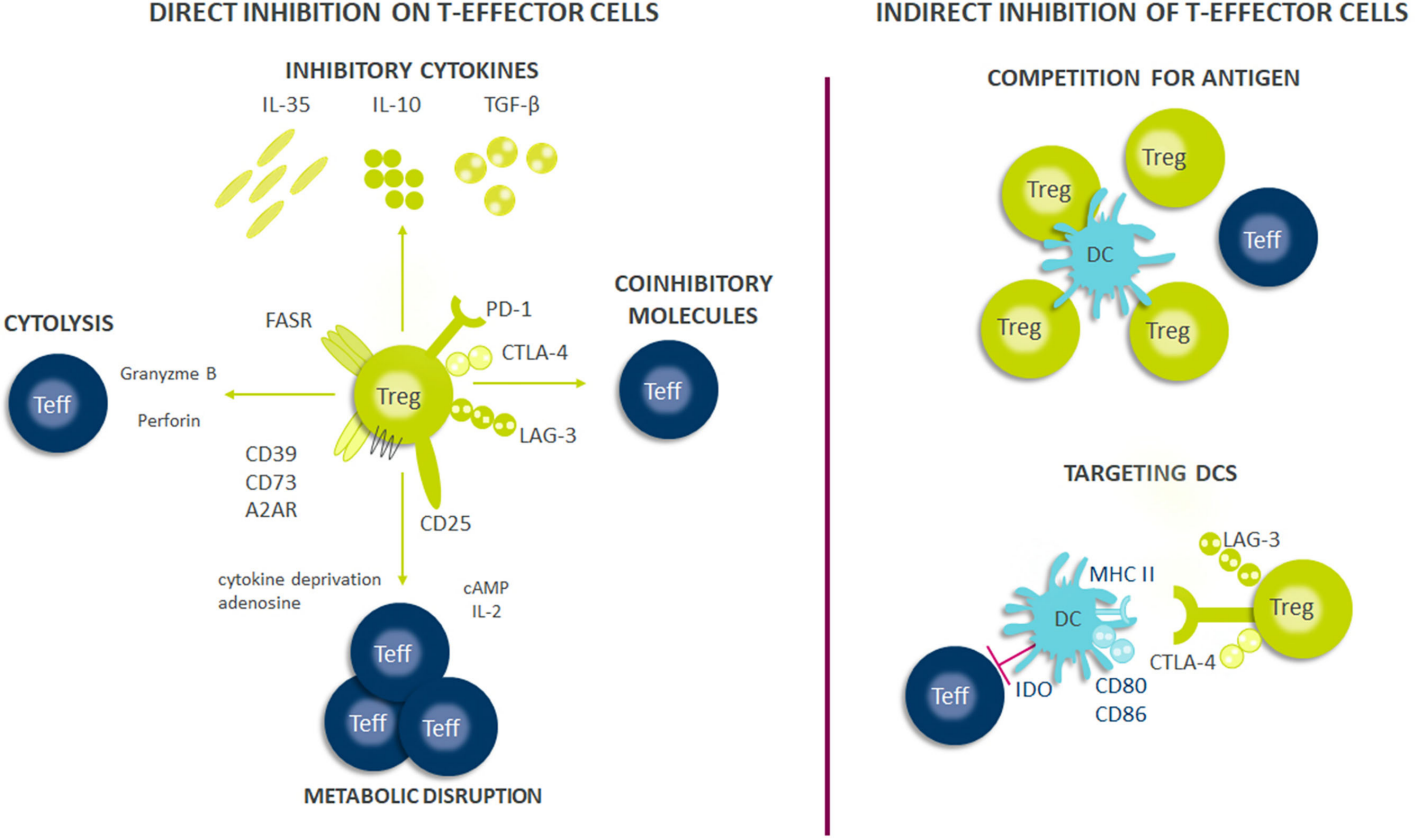
Suppressive mechanisms of regulatory T-cells (Tregs). Tregs exert their suppressive effects through several mechanisms, including direct and indirect inhibition of Teffs cells. Firstly, Tregs suppress effector cell responses through the release of soluble factors or through their consumption. Tregs secrete multiple inhibitory cytokines such as IL-10, IL-35, and TGF-β and can directly kill effector or antigen presenting cells through perforin and granzyme. Tregs also have high expression of CD25 (IL-2 receptor α-chain) and can consume IL-2 which is necessary for optimal Teff responses and survival. Further, the generation of adenosine from ATP/AMP which is metabolized by CD39/CD73, both of which are expressed on Tregs, results in Teff cell suppression from the induction of negative signaling. Contact dependent inhibition can have both direct and indirect effects on Teff cell responses. Tregs express FASR which can bind to FAS on Teffs and induce apoptosis. Further, Tregs express multiple immune check-point molecules such as LAG-3, CTLA-4, and PD-1 among others which inhibit Teff cells responses or drive dendritic cells (DCs) towards a tolerogenic phenotype. Tolerogenic DCs can produce indoleamine 2,3-dioxygenase (IDO) which exhausts T-cells because critical amino acids are depleted for Teff cell survival and can cause decreased expression of co-stimulatory molecules on their surface such as CD80/86. Finally, Tregs can also compete for antigens presented by DCs and thus limiting Teffs activation through antigen stimulation.

**Figure 2:**
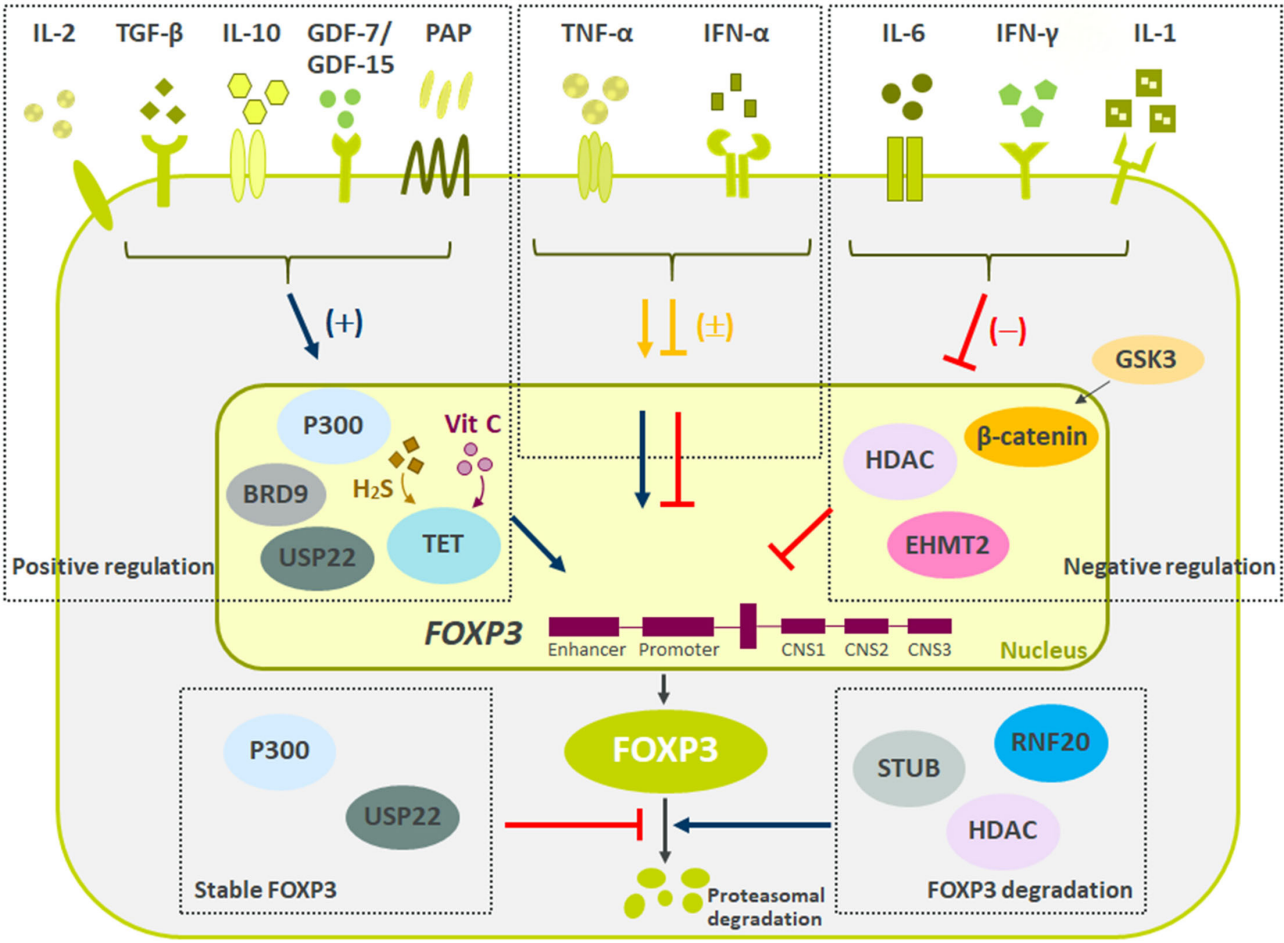
Summary of negative and positive regulators of FOXP3 expression. Reviewed here are recent molecules driving Tregs stability and function. Multiple secreted proteins have shown both positive (IL-2, TGF-β, IL-10, GDF7/15, PAP), negative (IL-6, IFN-γ, and IL-1), and mixed (TNF-α and IFN-α) effects on Treg biology. Besides secreted proteins, multiple pathways influencing FOXP3 methylation, ubiquitination and acetylation have recently been described. These changes to FOXP3 ultimately led to difference in Tregs suppressive capacity and stability. Finally, pathways regulating FOXP3 protein degradation are critical to maintain Treg cell lineage and function.

As Treg cells deploy more than a dozen molecular mechanisms to suppress immune responses (**Figure 1**), they have potential as multifaceted therapeutics in immune-mediated diseases. There is emerging evidence that Tregs reside in the non-lymphoid tissues and play a central role in regulating tissue homeostasis, repair and regeneration. Thus, the net impact of a Treg therapy on disease pathophysiology may exceed the efficacy of existing therapies. Early-phase clinical trials of Tregs therapy have shown good tolerability with clinical efficacy responses especially in transplant setting. However, transient nature of improvement has been observed in early trials with autologous *ex vivo* expanded Tregs. Current efforts are focused on determining the molecular mechanisms to optimize the survival, stability and suppressive functions of Tregs at steady state and in inflammatory milieu (**Figure 2**). The progress in the development of chimeric antigen receptors and in genome editing technology has facilitated the genetic optimization of CAR-T cell therapy for cancer. In the next decade, development in optimized CAR-Treg cells would lead to exciting new frontiers in the cell therapy field enhancing the specificity and functionality of Treg cells.

## Author Contributions

KJB, JL, and TO contributed to conception, literature evaluation and writing the review article. All authors contributed to review article revision, read, and approved the submitted version.

## Conflict of Interest

All authors were employed by the company AstraZeneca.

## Publisher’s Note

All claims expressed in this article are solely those of the authors and do not necessarily represent those of their affiliated organizations, or those of the publisher, the editors and the reviewers. Any product that may be evaluated in this article, or claim that may be made by its manufacturer, is not guaranteed or endorsed by the publisher.
